# “I was *that* close”: Investigating Players’ Reactions to Losses, Wins, and Near-Misses on Scratch Cards

**DOI:** 10.1007/s10899-015-9538-x

**Published:** 2015-04-11

**Authors:** Madison Stange, Candice Graydon, Mike J. Dixon

**Affiliations:** Department of Psychology, University of Waterloo, 200 University Avenue West, Waterloo, ON N2L 3G1 Canada

**Keywords:** Scratch cards, Gambling, Skin conductance, Arousal, Near-misses

## Abstract

While scratch cards are a popular, accessible, and inexpensive form of gambling, very little is known about how they affect and influence the player. This study sought to understand the physiological and subjective experience of scratch card play, with special emphasis on the effect of near-miss outcomes (i.e. uncovering two out of three “grand prize” symbols needed to win said prize), which are remarkably prevalent in scratch card games. Thirty-eight undergraduate students from the University of Waterloo each played two custom scratch card games and experienced three types of outcomes (losses, wins and near-misses) while their skin conductance levels (SCLs) and post-reinforcement pauses were recorded. Each participant also rated each outcome in terms of its subjective level of arousal, valence, and frustration. Our results indicate that players interpreted near-misses as negatively valenced, highly arousing, frustrating losses, and were faster to move onto the next game following this type of outcome than following winning outcomes. Additionally, near-miss outcomes were associated with the largest amount of change in SCLs as the outcome was revealed. This work has implications for the problem gambling literature as it provides evidence of the frustration hypothesis of near-misses in scratch cards, and is the first study to examine the physiological and psychological experiences of scratch card players.

## Introduction

Instant lottery games,^1^ which we will refer to as ‘scratch cards’, are a highly popular form of gambling activity that is readily available across Canada. For instance, individuals over the age of 18 can purchase scratch cards in Ontario at any one of nearly 10,000 lottery retailers (Ontario Lottery and Gaming Corporation 2014), including local pharmacies, supermarkets, convenience stores, and even some University store retailers. While these cards (in Ontario) can cost anywhere between $1 and $20, the vast majority of cards cost $5 or less.

Although scratch cards are typically grouped with lottery games at point of sale and within the gambling literature, some authors suggest that scratch cards represent a “harder” form of gambling due to their structural characteristics and the option of instantaneous and continual play (Griffiths and Wood 2001). As with slot machines, scratch card players know whether they have won or lost within seconds of initiating game play. It is this instantaneous nature of scratch cards that may make them distinct as a “lottery product”. Scratch card outcomes can be revealed in a second or two, whereas in traditional lottery games such as lottery draws, there is typically a lengthy waiting period before winning numbers are announced.

Research concerning scratch cards is sparse, and the literature that does exist focuses predominantly on the use of these products by youth. Despite the fact that lottery purchases are restricted to anyone under the age of 18 or 19 in Canada (depending on the province), numerous studies have shown alarming rates of youth participation in lottery forms of gambling, suggesting that youth and adolescents continue to gain access these products (Felsher et al. 2004a, b; Griffiths 2000). For instance, Felsher et al. (2004b) investigated lottery-playing behaviour amongst 1072 Ontario youth aged 10–18, and found that 54 % of participants self-reported having played scratch cards in the past 12 months. Furthermore, scratch cards were reported as the most popular form of lottery gambling amongst male and female youth, and were also rated as the most desirable gambling activity. Reasons for this preference included their low price point, high accessibility, and the immediate reinforcement afforded to the player. Griffiths (2000) reports a similar rate of scratch card ticket play (55 %), with 5 % of the sample of youths meeting DSM-IV criteria for pathological gambling on scratch cards.

In addition to youths purchasing their own lottery tickets, numerous sources report that parents purchase lottery tickets for their children (Campbell et al. 2011; Derevensky and Gupta 2001; Kundu et al. 2013). A study conducted by Kundu et al. (2013) found that high school students who were given lottery tickets as gifts were more likely to report signs of problem gambling and more permissive attitudes towards gambling than adolescents who had never received lottery tickets as gifts.

Although exceedingly popular, and potentially addictive, little is known about the characteristics of these games that may contribute to their particular appeal. Specifically, it is currently unknown how these types of games influence arousal, which has been identified as one of the most important reinforcers of gambling behaviour (Brown 1986). The present study sought to answer these questions and allow deeper insight into scratch card games and their characteristics.

In terms of their surface features scratch cards contain many comparable design elements and structural characteristics to those used in slot machines. Both frequently feature bright colours, attention-grabbing display features (e.g., metallic elements), and large jackpots/top prizes. Indeed Ariyabuddhiphongs (2011) encapsulates such similarities by suggesting that “scratch cards are essentially slot machines on paper.”

Importantly both slot machines and scratch cards feature a particularly intriguing type of outcome—the near-miss. In gambling parlance, a near-miss is defined as an outcome that is close to a jackpot, but falls just short (Reid 1986). In a typical scratch card game, if a player uncovers three identical jackpot symbols, they would win the grand prize. A scratch card near-miss involves players successively uncovering two of the three needed jackpot symbols within the symbol matrix, leaving the player with the feeling that they were “so close” to winning the jackpot.

As any scratch card player knows, scratch card near-misses are a very common outcome. For example, we purchased, five, two-dollar Cash For Life© scratch cards from an Ontario lottery retailer. These cards each contained three games (3 matrices of 6 or more symbols). In each game the player sought to uncover three identical symbols within the matrix to win the prize specified. The symbols were either dollar amounts (e.g., $2.00, $4.00, $6.00) or the word LIFE (denoting the top prize). Thus, uncovering three $4.00 symbols would lead to a $4.00 prize, uncovering three LIFE symbols would lead to the grand prize ($200 per week for life). In these cards a near-miss would be two instances of the word LIFE being uncovered within a given game. Although the exact frequency of LIFE near-misses over the population of printed tickets is (to our knowledge) not known, every single card in our small sample contained at least one near-miss featuring the top prize. One of the purchased cards had LIFE near-misses in all three games on the card.

Such near-misses are readily apparent to players because of low-level visual features. Within a game matrix, the majority of symbols are numerical amounts (e.g., $2.00, $4.00). By contrast, the grand prize symbol is the capitalized word “LIFE”. This symbol perceptually stands out from the other (numerical) symbols in the matrix. When two “LIFE” symbols occur in the matrix, they are likely to be noticed by players due to the combination of low-level features, and the fact that this symbol denotes the top prize. To appreciate how such near-misses may contribute to the allure of scratch cards, one must consider what is known about near-misses in another form of gambling—namely slot machine play.

On a three-reel slot machine, one spins the reels and hopes for the best-case scenario—that three identical jackpot symbols (e.g., 3 red 7 s) land on the payline. A classic near miss occurs when the first two reels stop with the red 7 s on the payline, with the third just above or below the payline (“7-7-X”). These outcomes are both highly salient and physiologically arousing (Clark et al. 2012; Reid 1986). Although near-misses and regular losses both result in no gain to the player, near-misses can still serve as strong reinforcers and even encourage slot machine play (Côté et al. 2003). For example, Clark et al. (2009) found that near-miss outcomes in a simplified slot machine game activated brain areas related to winning and reinforcement processing. Additionally, Clark et al. (2012) found that although near-miss outcomes were viewed as unpleasant by gamblers, their occurrence increased players’ urge to gamble.

Near-misses appear to encourage further play via frustration. Dixon et al. (2011) showed that near-misses triggered greater skin conductance responses (SCRs) during slots play than regular losses or small credit wins—a finding that aligns with research showing that SCRs are larger for frustrating than pleasant events (Lobbestael et al. 2008). Evidence for the frustration hypothesis also comes from studies that combine SCRs with post-reinforcement pauses (PRPs)—the time it takes a player to move from the outcome of one spin to the initiation of the next spin. Previous studies have shown that PRPs increase with win size (Delfabbro and Winefield 1999). This is thought to occur because a player pauses to internally celebrate the win; the bigger the win, the longer this pause is (Dixon et al. 2013). Dixon et al. (2013) showed that classic near-misses on a slot machine trigger high arousal (gauged by large SCRs), but very short PRPs (much shorter than the PRPs following wins), due to their frustrating nature.

Although traditional SCRs and PRPs are both measured following outcome delivery, Dixon et al. (2011) have shown that for near-misses, increases in arousal occur even before the outcome is known. Such increases in arousal are most likely brought about by the anticipation of a possible jackpot win that is triggered by the first two jackpot symbols falling on the payline. Thus to fully gauge the physiological effects of scratch card near-misses, researchers should assess the ramping up of arousal accompanying the appearance of the leading jackpot symbols prior to the outcome being revealed, as well as the arousal that occurs once the outcome is known (the frustration that accrues when the hopes of the player are dashed).

We used a combination of physiological and subjective measures to better understand the characteristics of scratch cards that may account for their particular allure. We hypothesized that, as with slot machine play, if players interpret a small win in scratch card games as a rewarding outcome, then they should show higher SCRs and longer PRPs compared to regular losses. Furthermore, after viewing each outcome, they should also rate the win as more subjectively arousing, more positively emotionally valenced, and less frustrating than the loss. For near-misses, if players become preferentially aroused by the appearance of the first two jackpot symbols then compared to both losses and wins, they should show elevations of skin conductance levels (SCLs) as anticipation builds. If after the outcome is revealed they consider the near-miss as a particularly frustrating kind of loss then they should show higher SCRs for these outcomes compared to either a loss or a small win. Importantly, players should show shorter PRPs following a near-miss than following a small win (mimicking the high-SCR small-PRP pattern shown by Dixon et al. 2013). Finally, they should report that near-misses are more subjectively arousing, more negatively emotionally valenced, and more frustrating than the regular losses.

## Methods

### Participants

Thirty-eight undergraduate students were recruited from the Department of Psychology’s Research Experience Group at the University of Waterloo. One participant was excluded from further analysis as they had taken a course that discussed research relevant to the present study. Another participant was excluded as they scratched their cards incorrectly (see procedure). One participant whose skin conductance data was out of range was excluded from only the SCR analyses. Finally, the winner of the top prize amount was not included in the analyses.

The remaining thirty-five participants (22 females) ranged in age from 18 to 40 (*M* = 21.26). Prescreening ensured that participants were all: (1) 18 years of age or older (the legal age to purchase scratch cards in Ontario), (2) experienced with scratch card type lottery games (3) not in treatment for problem gambling, and (4) and not currently in treatment for an anxiety disorder or taking any medication for an anxiety disorder. The latter restriction was used as some anxiolytic medication may interfere with the recording of skin conductance levels.

After giving informed consent, participants completed the Canadian Problem Gambling Index (CPGI; Ferris and Wynne 2001) electronically using Qualtrics online survey software. This questionnaire assesses a number of dimensions of gambling behaviour, including an individual’s involvement with gambling activities and problem gambling behaviour. Scores on the Problem Gambling Severity Index indicated that 25 participants were classified as non-problem gamblers; 6 participants as low-risk gamblers, 2 as moderate/at-risk gamblers, and 2 as problem gamblers. The CPGI was administered to characterize our sample. Due to low numbers of those with gambling problems, gambling status was not analyzed further.

Participants completed the study for course credit and had the opportunity to win monetary prizes based on the outcomes of their chosen scratch cards. All methods and procedures (described below) were approved by the University of Waterloo’s Office of Research Ethics.

### Materials

#### Scratch Cards

**Fig. 1:**
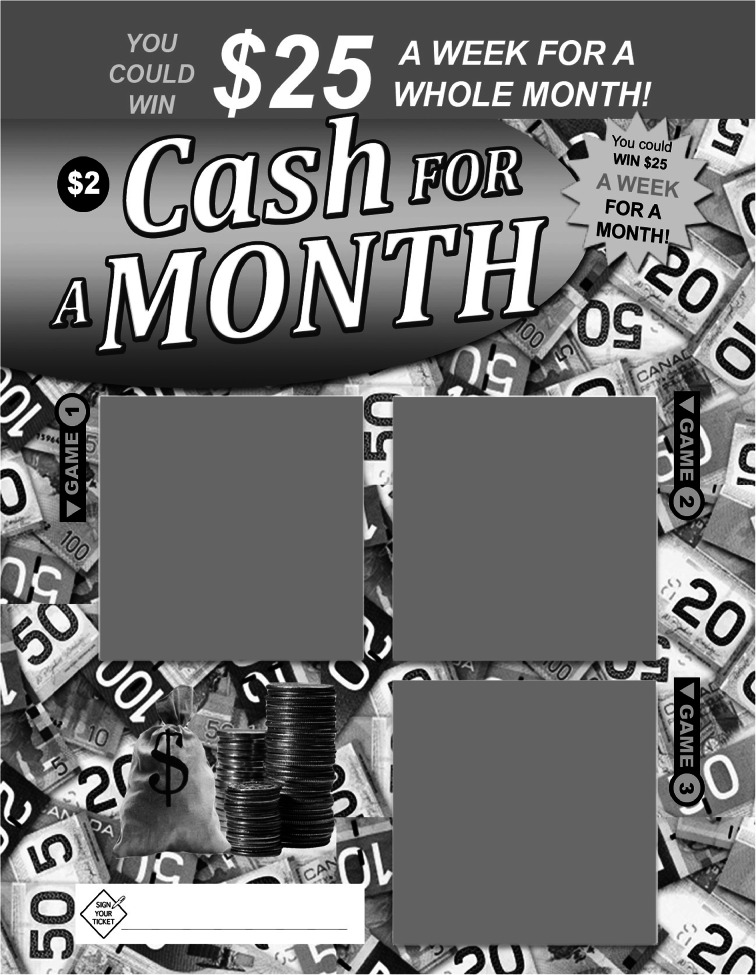
Our custom-made scratch card game, “Cash for a Month”

Participants played custom-made scratch cards (Fig. 1). These cards were designed to closely mimic the popular two-dollar version of the scratch card game, Cash For Life©, which is available for sale at many lottery retailers in Ontario. The cards used in the experiment were designed to emulate as closely as possible the Cash for Life© cards. Thus many of the same design features, and even a similar name—“Cash for a Month” were used. The featured top prize of our game was $25.00 CAD a week for a month ($100.00 CAD in total). These cards were printed on cardstock, and featured scratch-off game play areas that are functionally identical to those found in real scratch cards to ensure a realistic playing experience and the highest degree of ecological validity as possible.

#### Subjective Measures of Arousal, Valence, and Frustration

**Fig. 2:**
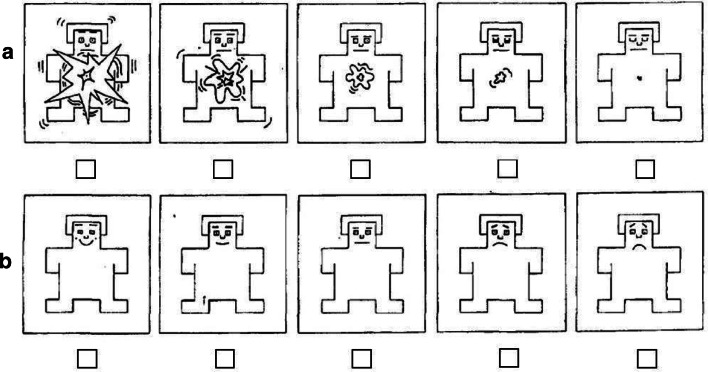
Self-Assessment Manikins (SAMs) that participants used to indicate their subjective arousal (**a**) and valence (**b**) in response to the three outcome types (loss, win, and near-miss)

Subjective measures of arousal, valence, and frustration were administered using paper and pencil questionnaires. These measures documented participants’ ratings of each type of game outcome (i.e. loss, small win, and near-miss) in terms of the three dimensions described above. Arousal and valence were measured using Self-Assessment Manikins (SAMs; Bradley and Lang 1994; Fig. 2). Level of frustration following each outcome was measured using a 5-point a Likert scale, where 1 was not at all frustrated and 5 was extremely frustrated.

### Apparatus

#### Scratch Card Display Case

**Fig. 3:**
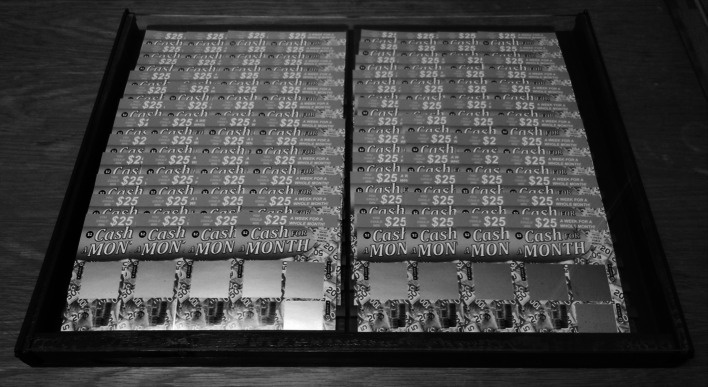
A display case similar to those found in Ontario Lottery Retailers was created to display the “Cash for a Month” cards

Participants selected two scratch cards that they wished to play from a custom made display case containing 96 custom made cards (players were instructed to select one card from the left tray, and a second from the right tray; Fig. 3). The display case resembled those found in many lottery retailers in Ontario to ensure a high level of ecological validity for the study.

#### Video Recording

Participants scratched both of their tickets on an angled scratching platform to ensure visibility to the participants and simultaneous video recording. Game play was recorded using the built in FaceTime HD (720p) camera available on the MacBook Pro laptop computer.

#### SCL Recording

SCLs were recorded using non-gelled passive skin conductance electrodes attached to the upper phalanges of the index and ring fingers of the participant’s non-dominant hand. The SCL electrodes were attached to a four channel ADinstruments Powerlab (model 4/35) equipped with a GSR amplifier. SCLs were time-locked to each outcome using the FaceTime video and LabChart 7.0 analysis software.

### Design

**Fig. 4:**
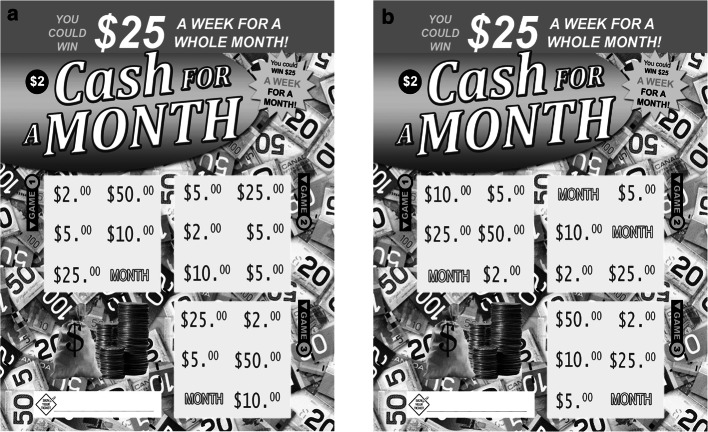
Two card designs of “Cash for a Month”

A within-subjects design was used where each participant experienced one small win (3 matching $5.00 symbols), one near-miss (players uncovered two of the three “MONTH” symbols needed to win the top prize), and four regular losses. Each participant played two scratch cards with three, 6-symbol games per card. Card “A” contained a loss, a small win, and another loss (See Fig. 4a). Card “B” contained a loss, a near-miss and another loss (See Fig. 4b). Each participant scratched one A-type and one B-type card (with order of cards counterbalanced across participants).

The rules of the scratch card games were based on those of Cash for Life©. If a player uncovered three identical symbols in one game, they won that prize. Therefore, losses consisted of six non-matching symbols; the small win consisted of three matching $5.00 symbols and three non-matching symbols; and the near-misses consisted of two matching “MONTH” symbols and four non-matching symbols. Non-matching symbols were taken from the prize structure of the original Cash for Life© scratch card game.

A total of 96 cards were presented in the display case. Players chose one from the left side of the case (containing 47 Card A, and 1 top prize card), and one from the right side (containing 48 Card B). Players were informed that one of the 96 cards contained the grand prize (i.e. had 3 “MONTH” symbols within one of the games). The location of this top prize card was randomized at the outset of the experiment.

### Procedure

Participants first read an information letter outlining the purposes and objectives of the study and completed a written consent form if they agreed to participate. Participants completed the CPGI using a laptop computer and Qualtrics online survey software. Following this, participants proceeded to the “lotto desk” containing the display case and scratch cards. Participants were told that they would be playing two scratch card games, and that the top prize for the game that they would be playing was $25 CAD a week for a month, which totals to a grand prize of $100 CAD. Participants were informed that there was one top prize card in the display case and that the odds of picking that card were approximately 1 in 100, as there were approximately 100 cards in the display case. The researcher then removed the trays containing the scratch cards from the display case and presented the cards to the participant. The participant was instructed to pick one card from each tray of cards.

Once the participant chose their cards, the researcher went over the rules of the scratch card game. Participants were shown, on an oversized example of a scratch card, that each card has three games, and that each game contains 6 symbols (this was emphasized by an overlaid 3 by 2 grid on one of the games on the example card). The researcher then explained that to win a prize, three symbols within one game had to match. Thus, to win the top prize, three “MONTH” symbols had to be uncovered within one game. The participant was then instructed to scratch each game in the same manner that they would read a paragraph (i.e., starting in the top left-most corner of the first row, finishing in the right-most corner of the bottom row). This was done to ensure consistency of outcome delivery between participants. Participants were asked to scratch each game completely prior to moving to the next game, and to scratch the games in order (scratching game 1, 2, and then 3; see Fig. 1). Participants were given the opportunity to ask the researcher any questions about the structure or rules of the game before the start of the experiment.

Participants then washed their hands, and were brought to the “playing area”, which consisted of a table set up for the purposes of scratching the cards and recording SCLs. The researcher then attached the skin conductance electrodes to the upper phalanges of the index and ring fingers of the participant’s non-dominant hand. The participant was instructed to keep this hand as still as possible while scratching the cards. The experimenter then placed one of the two chosen cards into a secure card holder to ensure that: (1) the card remained still while the participant scratched the card and (2) that the card was maximally visible for video recording of scratching. The scratching platform was angled at 30° to facilitate game play and recording.

Participants were then reminded to scratch the card (using a 10¢ Canadian coin; 18 mm diameter) one game at a time in a left-to-right fashion. After scratching their first card, the experimenter inserted the second card into the card holder of the scratching platform. Once participants completely finished scratching both cards, an occluder was placed over the card so that the participant’s view was restricted to a single game/outcome. Participants were asked to evaluate that outcome in terms of subjective arousal, valence, and levels of frustration they felt while playing that game. After rating one game/outcome, a new occluder revealed different outcome and the process was repeated until the participant had rated a loss, a win and a near miss. The orders with which they viewed and rated the outcomes were completely counterbalanced (6 possible orders). Participants were then debriefed, given their winnings, feedback about the study, and two responsible gambling brochures.

## Results

### Data Reduction

In scratch card games, outcomes (i.e., losses, wins, and near-misses) are only known when the last symbol in a game is uncovered. Pre-outcome changes in SCLs over the course of uncovering the various symbols were assessed by defining a window that began when the participant scratched the first symbol in a given game, and ended when they finished scratching the last symbol in that game. Change scores were the maximum within this window (the peak), minus the value at the beginning of the window. A square root transformation was applied to this data to minimize skew within the distribution of the data (Dawson et al. 2007).

Post-outcome SCRs were calculated in a similar fashion except the window was a 3-s window that began 1 s after the last symbol in the card was fully uncovered. SCRs were the square root of the maximum within this window (the peak), minus the value at the beginning of the window. PRPs were calculated by extracting the temporal distance (in seconds) between the last symbol in a game being uncovered, and the scratching of the first symbol in the next game.

Participants’ data for each dependent variable (SCLs, SCRs, PRPs, and subjective ratings of arousal, valence, and frustration) was first submitted to an outlier rejection procedure using a cutoff of SD = 3. The remaining data for each dependent variable were submitted to separate one-way repeated measures analyses of variances (ANOVAs), with game outcome (loss, near-miss, win) as the repeated measures factor. Any significant main effects were further analyzed using paired samples *t* tests (equivalent to Fisher’s least significance differences test).

### Pre-outcome Skin Conductance Changes

**Fig. 5:**
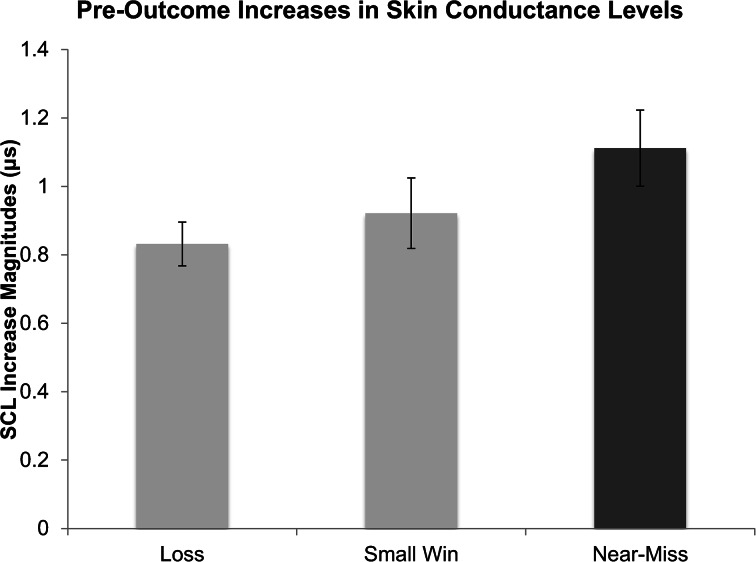
Pre-outcome skin conductance levels (SCLs) leading up to wins, losses, and near-misses, as a function of outcome. Error bars are ± 1 SE

Given that each participant experienced four losses (compared to one win and one near-miss), we averaged the SCLs for the four losses. Thus, the mean was used to represent any changes in SCLs as the symbols were uncovered during these outcomes. Shown in Fig. 5, near-misses were associated with the largest changes in SCL, followed by wins, then losses showing the smallest change. These differences were verified by a repeated measures analysis of variance conducted on average SCL change magnitudes (including change values of zero). This analysis revealed a significant main effect of outcome *F*(2, 66) = 4.92, *p* = .01. Fisher’s LSD post-hocs revealed that near-misses (*M* = 1.11, *SD* = .65) were associated with larger SCL increases than regular losses (*M* = .83, *SD* = .37), *t*(33) = 2.98, *p* = .005, and larger increases than small wins (*M* = .92, *SD* = .60), *t*(33) = 2.09, *p* = .045. Wins were not significantly different from losses *t*(33) = 1.02, *p* = .32.

### Post-outcome Skin Conductance Responses

**Fig. 6:**
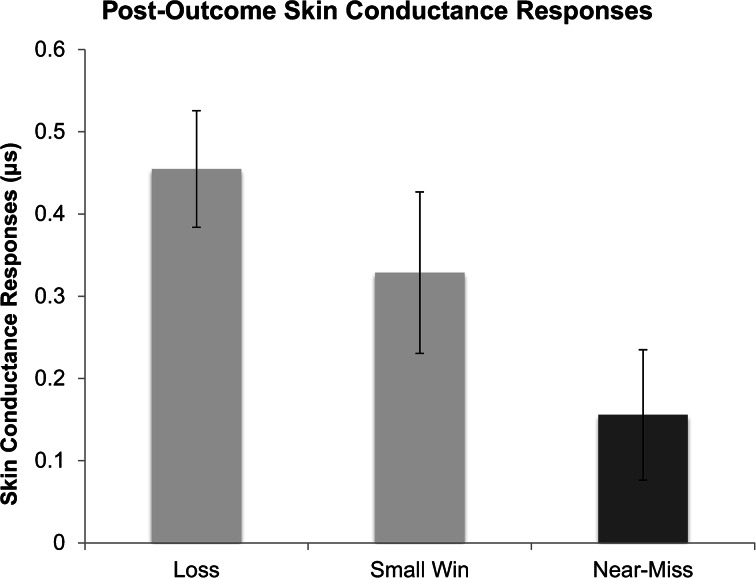
Post-outcome skin conductance responses (SCRs) following wins, losses, and near-misses, as a function of outcome. Error bars are ± 1 SE

Given that each participant experienced four losing outcomes, these four losses were averaged to give an aggregate value of SCRs during losses. Participant’s SCRs differed depending on outcome type, *F*(1.576, 48.862) = 5.50,^2^ *p* = .011 (Fig. 6). Participants showed higher SCRs following losses (*M* = .45, *SD* = .40) than near-misses (*M* = .15, *SD* = .45), *t*(31) = 3.76, *p* = .001. Participants did not differ, however, in their SCRs following losses and wins (*M* = .33, *SD* = .55), *t*(31) = 1.65, *p* = .11, or following wins and near-misses, *t*(31) = 1.55, *p* = .13.

### Post-reinforcement Pauses

**Fig. 7:**
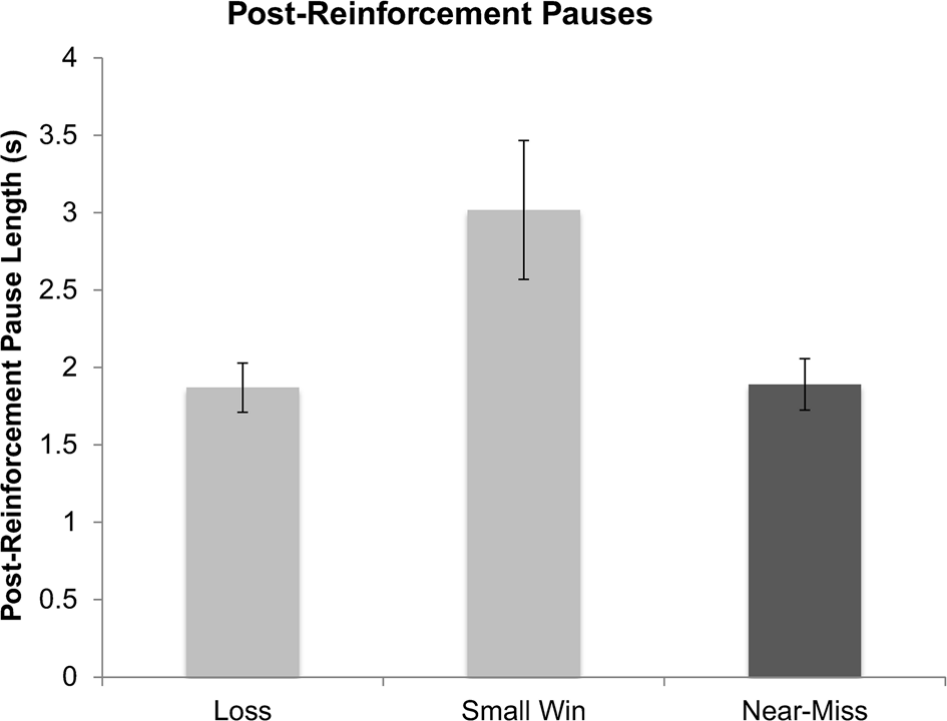
Post-reinforcement pause (PRP) length following each outcome type (measured in seconds). Error bars are ± 1 SE

Participants’ post-reinforcement pauses differed depending on outcome type, *F*(1.24, 35.96) = 6.04,^3^ *p* = .014 (Fig. 7). Participants showed shorter PRPs following near-misses (*M* = 1.89, *SD* = .91) than wins (*M* = 2.98, *SD* = 2.46), *t*(29) = −2.54, *p* = .017, and shorter PRPs following losses (*M* = 1.89, *SD* = .87) than wins, *t*(29) = −2.56, *p* = .016. Participant’s PRPs following losses and near-misses did not differ, *t*(29) = −.031, *p* = .98.

### Subjective Measures of Arousal, Valence, and Frustration

**Fig. 8:**
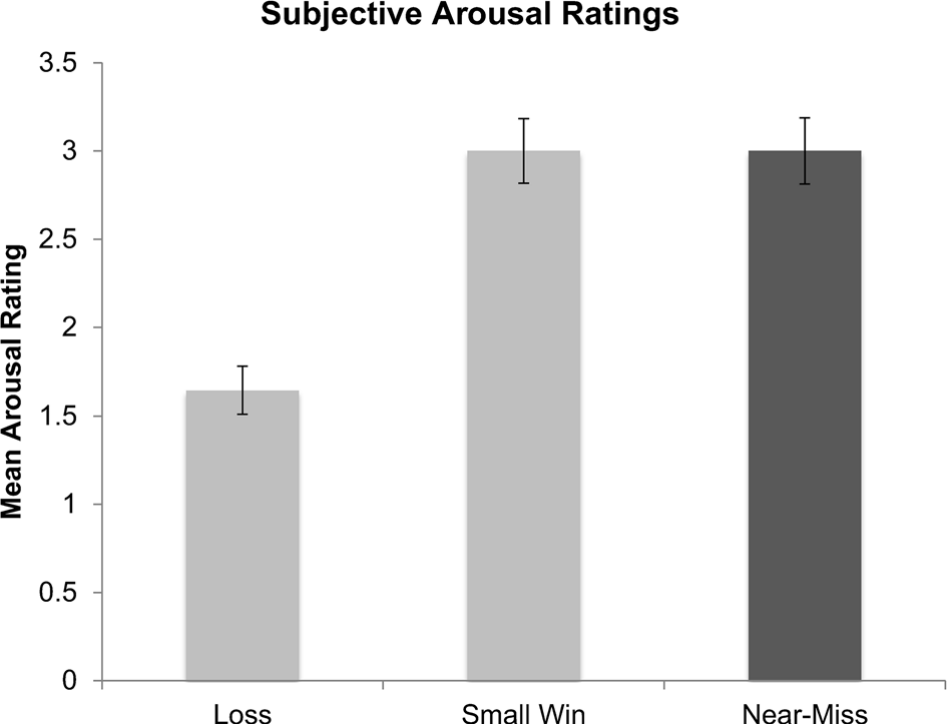
Subjective arousal ratings for each outcome type (loss, win, and near-miss). Error bars are ± 1 SE

Participants’ ratings of subjective arousal were different depending on outcome type, *F*(2, 60) = 34.77, *p* < .001 (Fig. 8). Participants rated losing outcomes (*M* = 1.66, *SD* = .80) as less arousing than near-miss outcomes (*M* = 2.94, *SD* = 1.13), *t*(33) = −7.45, *p* < .001, and rated losing outcomes as less arousing than winning outcomes (*M* = 3.06, *SD* = 1.08), *t*(31) = −7.27, *p* < .001. There was no significant difference between participants’ arousal ratings of near-misses and wins, *t*(30) = .00, *p* = 1.0.

**Fig. 9:**
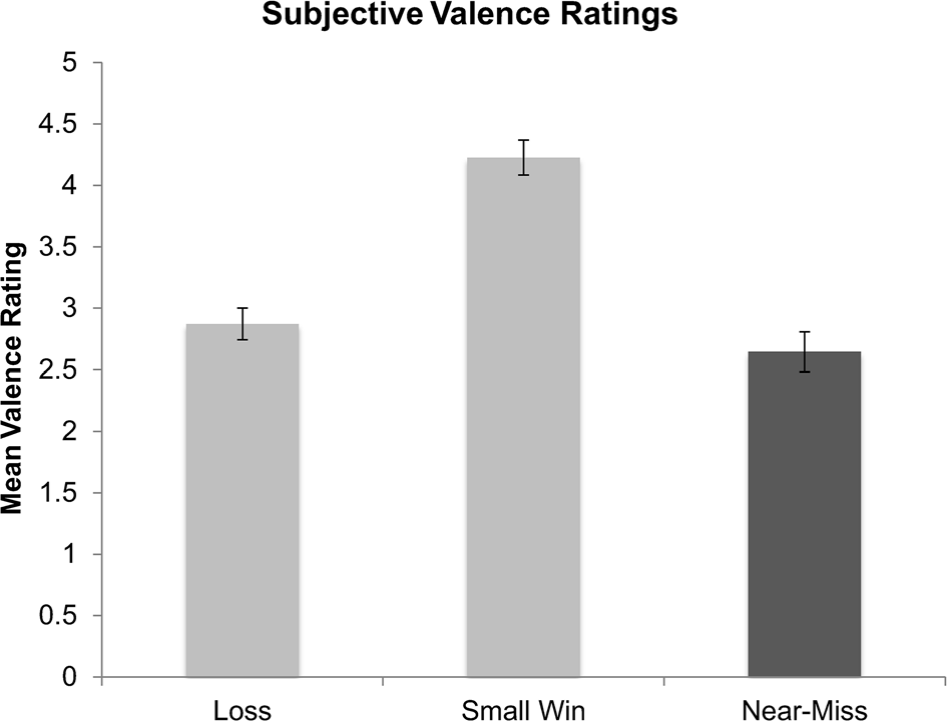
Subjective valence ratings for each outcome type; high values indicate more positive affect and low values indicate more negative affect associated with the outcome. Error bars are ± 1 SE

Participant’s ratings of subjective valence were different depending on outcome type, *F*(2, 60) = 40.75, *p* < .001 (Fig. 9). Participants’ ratings of near-misses (*M* = 2.65, *SD* = .92) as being lower in valence (more negative) than losses (*M* = 2.91, *SD* = .75) fell just short of significance, *t*(33) = −1.95, *p* = .059. Participants rated near-misses as more negative than wins (*M* = 4.25, *SD* = .80), *t*(30) = −7.48, *p* < .001, and losses were rated as more negative than wins, *t*(31) = −6.89, *p* < .001.

**Fig. 10:**
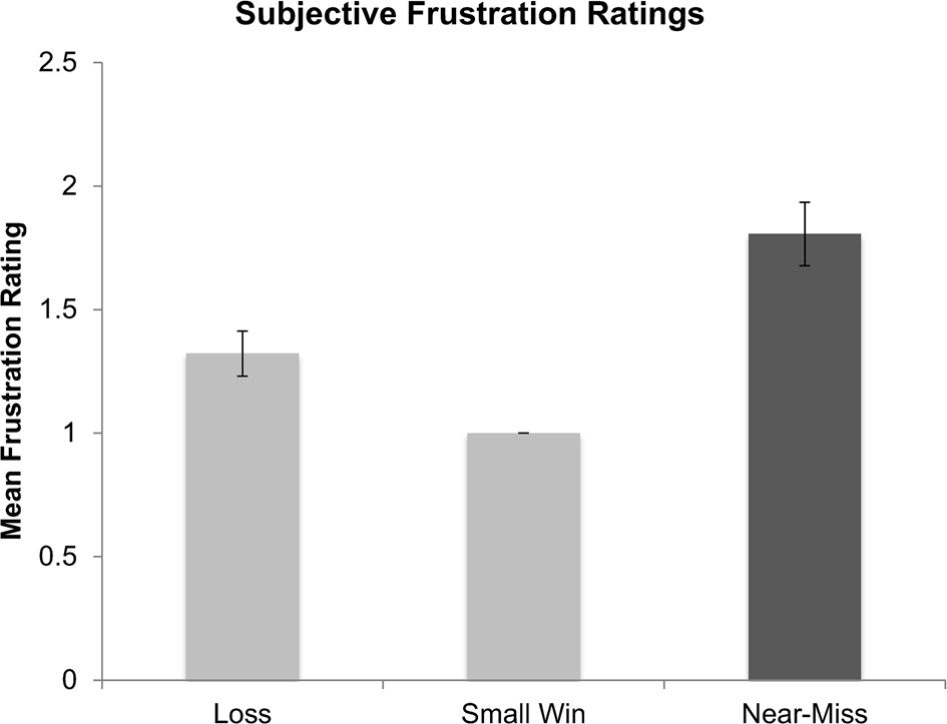
Subjective ratings of frustration for each outcome type on a scale from 1 (not at all frustrated) to 5 (extremely frustrated). Error bars are ± 1 SE

After outliers were removed, all participants gave a rating of minimum frustration following the winning outcome (Fig. 10). The lack of variance in this condition precluded conducting an analysis of variance. Non-parametric (Wilcoxon’s matched-pairs signed-ranks test) analyses indicated that near-misses (*M* = 1.85, *SD* = .74) were rated as more frustrating than losing outcomes (*M* = 1.40, *SD* = .55), *Z* = −3.77, *p* < .001, and more frustrating than wins (*M* = 1.0, *SD* = .000), *Z* = −4.18, *p* < .001. Additionally, participants rated losses as more frustrating than wins, *Z* = −3.05, *p* = .002.

## Discussion

In this study, participants experienced three different kinds of outcomes within two scratch card games: four losses, one small win, and one near-miss. Our hypotheses were based on the frustration account of near-misses in slot machines, which theorizes that players experience a build up in anticipation when they see two jackpot symbols, but then react with frustration when the third symbol fails to complete the sequence. From an objective monetary standpoint, these outcomes are technically no different from regular losses. Nevertheless, we expected that they would produce high physiological and subjective arousal in our participants, and cause players to move on to the next available game more quickly than following a positive outcome. Furthermore, if near-misses were indeed particularly frustrating losses, they should lead participants to subjectively rate these outcomes as being negative in emotional valence, and high in frustration. In contrast, positive outcomes also produce high physiological arousal due to their rewarding properties, but (unlike near-misses) elicit a longer pause between games as the player takes time to internally celebrate their win. Additionally, if these outcomes are indeed experienced as positive, rewarding events, we would expect them to trigger high ratings of subjective arousal, positive ratings of emotional valence, and low ratings of frustration.

The majority of our hypotheses were confirmed. Wins were rated as equally high in arousal as near-misses, the lowest in terms of frustration, and the highest (most positive) in emotional valence. Additionally, wins were found to elicit longer PRPs than all other outcomes. Most importantly, near-miss outcomes in scratch cards were rated as the most frustrating and the most negatively valenced of all three outcome-types. Near-misses were also rated as more subjectively arousing than losing outcomes, and equally as arousing as winning outcomes. As players uncovered the symbols in each game, there was a greater rise in SCLs as players uncovered the two “MONTH” symbols, than in either the win, or regular loss condition. Crucially, the PRPs following near-miss outcomes were shorter than those following winning outcomes, and equally as short as losing outcomes. This pattern of high arousal while uncovering the symbols, high ratings of frustration and negative valence as their hopes were dashed, and short PRPs indicating a wish to move quickly on to the next game provides converging evidence in support of the hypothesis that players respond to near-miss outcomes in scratch cards as frustrating losses, both in their retrospective subjective ratings, and also in their playing behaviours.

The only measure that violates an otherwise clear picture of how players responded to the various scratch card outcomes was the post-outcome SCR data. Contrary to our hypotheses, losses triggered significantly larger SCRs than near-misses (and marginally more than wins). This pattern is the *exact opposite* of what one would expect, based on research measuring physiological responses to gambling outcomes (Clark et al. 2012; Dixon et al. 2011, 2013). The pattern of data is also misaligned with the subjective arousal ratings, and perhaps most importantly is exactly opposite to the pattern observed when we measured the pre-outcome changes in SCLs during the uncovering of the symbols during the game. The most likely explanation is that the significant SCL changes prior to outcome delivery contaminated the measurement of post-outcome SCRs. Succinctly, the already high SCL levels triggered by the onset of the two LIFE symbols created a spuriously high baseline value at the beginning of the post-outcome SCR window from which further elevations were unlikely to be seen. This resulted in artificially small post-outcome SCRs following near-misses. By contrast, for losses, the small elevations in SCLs prior to outcome delivery rendered a low baseline value at the beginning of the SCR window with plenty of room to show SCRs due to even the mild frustration associated with the regular losses.

Given the nonsensical nature of the post-outcome SCR results when contrasted with the extant literature and the participants own subjective reports, we put greater weight on the rise in SCLs accompanying the uncovering of the two “MONTH” symbols, and suggest that the post-outcome SCRs are an artifact of these pre-outcome changes in arousal. In sum, after eliminating from consideration the post-outcome SCRs, we are left with subjective, physiological and behavioural data that all converge to suggest that near-misses are treated very differently from other outcomes. They appear to be a particularly arousing, frustrating loss, that we know from the gambling literature impacts the urge to continue gambling (Clark et al. 2012) and play durations (Côté et al. 2003).

This research, coupled with previous studies of youth use of scratch card games, paints a worrisome picture about scratch card use. In Ontario, scratch cards are advertised on national TV channels, billboards, and other forms of media. These products are readily available and widely purchased on university campuses (i.e., the participants in this study). Despite the somewhat lax attitude towards them and the tendency to group them with other “soft” forms of gambling such as lotteries, scratch cards share some concerning overlap with “hard” forms of gambling such as slot machines. Foremost among these shared features is the near-miss. In both slot machines and scratch cards, near-misses trigger increases in physiological and subjective arousal, frustration, and negative valence. Thus it would not be surprising if these near-misses in scratch cards also increase desire or urge to gamble as they do in slot machines. This is a troubling prospect considering the existing availability and relatively benign reputation of these games. Further research is needed to confirm whether scratch card near-misses do indeed increase the urge to gamble, and whether such urges translate to spending more money than intended on purchasing further scratch cards. The results of this study, however, suggest that perhaps it is time to reconceptualize scratch card games as a “harder” form of gambling (Griffiths and Wood 2001). The results of this study combined with the knowledge that scratch card games afford players the opportunity for continuous and repeated play emphasizes the need for the re-evaluation of these products’ potential for misuse.

Though not historically an area of extensive focus in gambling research, lottery games, in particular scratch cards and other instant games, are an important avenue of exploration. With youth accessing these products at alarming rates and their clear surface similarities to slot machines and other gambling forms, understanding how scratch cards emotionally, physically, and behaviourally affect the player is paramount. To our knowledge, this study is the first investigation of the physiological, behavioural, and subjective effects of experiencing varying outcomes on a scratch card game. Importantly, this is also the first study to explore the effects of near-miss outcomes in these games, despite the fact that near-misses on slots are known to trigger large physiological responses in the player and increase player’s urge to gamble (Clark et al. 2012). The converging evidence that players react to scratch card near-misses with the same pattern of high arousal, high frustration and short PRPs, as seen in slots players’ reactions to near-misses, highlights the potential dangers of this form of gambling.

Future directions for this line of research includes utilizing other physiological measures to understand the physical effects that these games have on the player. Specifically, heart rate deceleration has been used as an index of arousal (Dixon et al. 2011; Clark et al. 2012), and this measure could be useful in gaining a clearer picture of the physiological impact of these games. Additionally, introducing a measure of participant-applied force to a sensor following each outcome would allow for a physiological measure of frustration following the different outcome types (Dixon et al. 2014). It is also important to remember that when it comes to technological advances, gambling is not unaffected. Recently there have been more and more mobile applications allowing users access to virtual scratch card games, leading to questions of whether these virtual games would elicit responses in the player which are similar to the results of this study. These are just some of the possible ways in which scratch cards can be further explored. While we are only beginning to understand the structural characteristics of scratch cards and how they influence and affect the player, it is certain that there is much more concerning these games that remains to be uncovered.

## Footnotes

1 As termed by the Ontario Lottery and Gaming Corporation (OLG).

2 Assumption of sphericity violated (Mauchly’s test: *X* ^*2*^(2) = 9.40, *p* = .009; Greenhouse–Geisser correction applied).

3 Assumption of sphericity violated (Mauchly’s test: *X* ^*2*^(2) = 26.59, *p* < .001; Greenhouse–Geisser correction applied).
